# Physicochemical Properties of Safflower (*Carthamus tinctorius* L.) Seed Meal Protein and the Effects of pH and Ionic Strength on Its Functional Characteristics

**DOI:** 10.3390/foods15030593

**Published:** 2026-02-06

**Authors:** Yanling Yang, Yucheng Bai, Xiaoling Xie, Bingjing Li, Liping Luo, Qian Zhang, Cheng Luo, Wenxing Nie, Rui Qin, Hong Liu, Jiao Liu, Hongzao He

**Affiliations:** 1 Guizhou Institute of Biology, Guizhou Academy of Sciences, Guiyang 550009, China; 2021110338@mail.scuec.edu.cn (Y.Y.);; 2 Hubei Provincial Key Laboratory for Protection and Application of Special Plant Germplasm in Wuling Area of China, College of Life Sciences, South-Central Minzu University, Wuhan 430074, China; 3 Guizhou Academy of Testing and Analysis, Guizhou Academy of Sciences, Guiyang 550014, China

**Keywords:** safflower seed meal, protein isolate, functional, pH, ionic strength

## Abstract

In recent years, plant proteins recycled from agricultural waste have gained increasing attention in food manufacturing due to the relatively low environmental and economic cost. Safflower (*Carthamus tinctorius* L.) is an important edible oil crop which generates a large amount of seed meal as by-products. Thus, this study aimed to investigate the properties of proteins extracted from the safflower seed meal as food materials. The physicochemical properties and functional characteristics of safflower seed meal protein (SMP) were analyzed at different NaCl concentrations and pH. Results showed that the extraction rate of SMP is 55% and SMP contains 86% protein with an isoelectric point of 4.5. The molecular weight of proteins in SMP predominantly ranged from 10 to 43 kilodaltons (kDa), with a maximum weight loss temperature of 317 °C. Glutamic acid exhibited the highest, while lysine served as the primary limiting amino acid. All seven essential amino acids were present except for tryptophan, which was not included in the testing scope. Additionally, SMP exhibited its highest solubility (59.55%) and emulsifying capacity (62.63 m^2^/g) at pH 11, and its highest foaming capacity (70.67%) at pH 9. The highest solubility (41.56%) was observed at 1 mol/L NaCl; the highest emulsifying capacity (16.88 m^2^/g) was observed at 0.6 mol/L NaCl; and the highest foaming capacity (90.67%) was observed at 0.7 mol/L NaCl. This study demonstrates that SMP has excellent nutritional value and a variety of functional properties, making it a promising plant-based protein source for the food processing industry. Subsequent processing involving adjustment to a high pH and increased NaCl concentration can help SMP to exhibit its processing characteristics.

## 1. Introduction

In comparison with animal-derived proteins, plant-derived proteins exhibit a lower fat and cholesterol content and a high fiber content [1,2]. It has been demonstrated that the ingestion of plant proteins can reduce the amount of fat consumed and thus lower the risk of chronic metabolic diseases such as hyperlipidaemia and obesity [3,4]. Moreover, increasing the consumption of plant-derived protein foods has the potential to alleviate the pressures on the environment from livestock products, thereby offering sustainability at both environmental and societal levels [5,6,7]. Therefore, the food industry and scientists have been exploring various plant protein sources to meet the growing demand. In this context, agricultural and food production by-products representation has been regarded as a promising but under-utilized resource for sustainable protein supply [8,9].

Safflower (*Carthamus tinctorius* L.) is an annual plant that belongs to the Asteraceae (Compositae) family [10]. Safflower is indigenous to South Asia and it is now cultivated on a wide scale in the United States, Russia, India and China [11,12]. Safflower is an economically viable crop with multiple applications in edible oil, food additives and pharmaceutical production [11]. The economically valuable components of the plant include the tubular flowers, stems and leaves, and seeds [13,14]. Safflower seed oil contains a high level of essential unsaturated fatty acids and vitamins [15]. Among all known oil crops, safflower seed boasts the highest linoleic acid content, thus earning it the title “King of Linoleic Acid” [15,16]. Safflower seed meal is a by-product of safflower seed oil extraction, containing approximately 20–50% protein [17]. However, until now, proteins from safflower seed meal have been greatly under-utilized. The predominant ways include feed supplementation in livestock farming [18,19] or just being discarded as agricultural waste [20], which not only cause a waste of edible protein resources but also has adverse effects on the environment. Safflower seed meal protein (SMP), obtained through a process of extraction and purification, possesses both excellent nutritional value and rich functional properties. Studies have shown that the emulsifying and emulsion stability, as well as foaming and foam stability, of SMP in some circumstances could exceed that of soy protein [17,18]. Therefore, it could be regarded as a promising edible protein resource with significant potential.

At present, various plant-derived proteins have been widely used in the food processing industry, such as soy protein, pea protein, and corn protein [21]. They not only provide essential nutrients but also enhance the flavor, texture, and overall quality of foods [22,23]. However, both soy protein and pea protein carry a distinct beany odor, a sensory flaw that makes them difficult for most consumers to accept [24]. Corn protein, meanwhile, suffers from an inherent weakness of extremely poor water solubility, limiting its application scenarios. The physicochemical properties and functional characteristics of proteins are pivotal factors influencing their application potential in the domain of food processing [25,26]. The functional properties of proteins are subject to the influence of intrinsic factors and a multitude of environmental factors, including the pH and ionic strength of protein solutions [27,28]. Compared to these commonly used plant proteins, SMP has been less studied, resulting in its under-utilization within the food processing industry.

Therefore, this study aimed to first establish an alkali-solubilization and subsequent acid precipitation method to isolate SMP. Then, the nutritional value and physicochemical properties of SMP were characterized through electrophoresis, amino acid analysis, and other techniques. A systematic investigation on SMP functionality was conducted on the effects of varying NaCl concentrations and pH conditions on the functional characteristics of SMP, aiming to provide a theoretical basis for the high-value utilization of SMP.

## 2. Materials and Methods

### 2.1. Raw Materials and Pre-Treatment

Safflower seed meal harvested in 2021 was provided by Xinjiang Sunshine Greenland Agricultural Technology Co., Ltd. (Alashankou City, Bortala Mongol Autonomous Prefecture, Xinjiang, China) as a by-product of safflower seed oil production. Safflower oil was extracted by cold pressing the seeds after they were hulled and de-skinned, leaving a residual oil content of approximately 6%. The first step was to degrease the meal. Safflower seed meal was dried in a 40 °C oven until the weight became stable, and then was sifted through a 60-mesh screen. The meal was weighed, placed into a folded filter paper cylinder, and sealed. The sealed cylinder was then positioned in the Soxhlet extraction apparatus. Petroleum ether was added at a ratio of 1:15 (*w*/*v*), and the water bath temperature was set to 45 °C for refluxing for 24 h. Following the degreasing process, the sealed filter paper cylinders were left to air-dry naturally until a constant weight was achieved. The materials were then subjected to a sifting process through a 60-mesh screen, after which they were sealed and stored for future use.

### 2.2. Determination of the Isoelectric Point of Safflower Seed Meal Protein Isolate (SMP)

Two grams of degreased safflower seed meal was accurately weighed and added to 39.6 mL of ultrapure water at a ratio of 1:19.8 (*w*/*v*). The pH of safflower seed meal aqueous solution was adjusted to 10.5 with 1 M NaOH, and the bottle mouth was sealed with fresh-keeping film or sealing film. The mixture was heated in a water bath at 25 °C and magnetically stirred for 90 min. It was then centrifuged at 4 °C and 6000 rpm for 10 min, with the precipitate discarded and the supernatant retained. The pH of the supernatant was adjusted to 2.2, 2.5, 3.3, 3.5, 4.0, 4.5, 5.0, 5.5, 6.0 and 6.5 using 1 M HCl. Then, 200 μL of each pH sample was taken and centrifuged at 6000 rpm for 10 min. The protein concentration of the supernatant was determined by Coomassie Brilliant Blue method.

### 2.3. Extraction of SMP

In the present study, SMP was extracted by the alkaline extraction and acid precipitation methods, with slight modifications [24,29]. Degreased safflower seed meal powder was dispersed in distilled water at a weight ratio of 1:19.8, and pH was adjusted to 10.5 with 1 M NaOH. The suspension was stirred at 25 °C for 1.5 h, and then centrifuged at 4 °C at 4600× *g* for 10 min. The pH of the supernatant was adjusted to 4.5 with 1 M HCL and centrifuged at 4 °C at 4600× *g* for 10 min. The precipitate was collected and washed with distilled water, and then re-suspended in distilled water with pH adjusted to 7.0 by 1 M NaOH. The SMP solution was dialyzed at 4 °C for 48 h and then freeze-dried. The protein content of defatted safflower seed meal and SMP was determined using the Kjeldahl method. The calculation formula for the SMP extraction rate is as follows:

(1) $$Extraction\;rate\;\left(\%\right)=\frac{m_{1}\times \omega_{1}}{m_{2}\times \omega_{2}}\times 100\%$$

where *m*_1_ is the mass of SMP (g) and *ω*_1_ is the protein mass fraction of SMP (%); *m*_2_ is the mass of safflower seed meal (g) and *ω*_2_ is the protein mass fraction of safflower seed meal (%).

### 2.4. Sodium Dodecyl Sulfate-Polyacrylamide Gel Electrophoresis (SDS-PAGE)

Firstly, the pH of protein suspension was adjusted to 3.0, 4.0, 5.0, 6.0, 7.0, 8.0, 9.0, 10.0, 11.0, and 12.0 with 1 M NaOH and 1 M HCl. The mixture was thereafter centrifuged at 4 °C and 6000 r/min for 10 min, after which the supernatant was retained. The protein content was measured by the Coomassie Brilliant Blue method. Samples were diluted to an appropriate concentration (10 μg final loading volume) for electrophoresis. SDS-PAGE was performed using NuPAGE^®^ 10% Bis-Tris precast gel (Invitrogen, Thermo Fisher Scientific, Carlsbad, CA, USA) at 200 V and 100 mA. The gel was subjected to a 30 min staining process using a Coomassie Brilliant Blue staining solution, composed of a 3:1:6 (*v*/*v*/*v*) ratio of ethanol: acetic acid: water, with 0.13% Coomassie Brilliant Blue R250. The destaining solution was composed of a 3:1:6 (*v*/*v*/*v*) mixture of ethanol, acetic acid and water. An SDS-PAGE pre-stained ladder ranging from 180 to 10 kDa was used as a standard marker.

### 2.5. Amino Acid Composition Analysis

The amino acid composition of degreased safflower seed meal and SMP was analyzed using a high-speed amino acid analyzer (L-8900, Hitachi, Minato-ku, Tokyo, Japan). About 1–5 g of the sample was placed into a 20 mL hydrolysis tube, and 16 mL of 6 mol/L hydrochloric acid solution was added. The tube was vacuum-degassed for 30 min, filled with nitrogen, and then sealed. Subsequently, the sample was hydrolyzed at 110 °C for 22–24 h. After hydrolysis, the tube was removed, cooled, and opened. The contents were non-destructively transferred to a 50 mL volumetric flask using deionized water and then diluted to volume. Exactly 1 mL of the hydrolysate was transferred into a vial, and the sample was deacidified and dried under vacuum. Then, water was repeatedly added to the vial followed by drying to ensure complete deacidification. The treated sample was set aside for later use. Prior to instrument analysis, 1 mL of 0.02 mol/L hydrochloric acid solution was added to the vial to ensure complete dissolution of the sample. Finally, the solution was filtered through a 0.22 µm aqueous phase filter membrane before instrument analysis was performed. The high-speed amino acid analyzer conditions are as follows:

1. Column: 2622# PH ion-exchange column, 4.5 × 60 mm;
2. Column temperature: 57 °C;
3. Post-column derivatization: Derivatization reaction temperature 135 °C;
4. Injection volume: 20 μL;
5. Flow rate: Derivatization reagent 0.35 mL/min; buffer: 0.4 mL/min.

The amino acid score (AAS) is calculated using the following formula:

(2) $$\mathrm{AAS}=\frac{Essential\;Amino\;Acid\;Content\;in\;the\;Sample}{\;Essential\;Amino\;Acid\;Content\;in\;the\;FAO/WHO\;Scoring\;Model}\times 100\%$$

### 2.6. Thermalgravimetric Analysis (TGA)

For thermogravimetric analysis (TGA), 5–10 mg of SMP powder was sealed in an aluminum pan. Using a thermogravimetric analyzer (TG209 F3, NETZSCH-Gerätebau GmbH, Selb, Bavaria, Germany), the sample was heated from 40 °C to 600 °C at a heating rate of 10 °C/min under a nitrogen flow rate of 50 mL/min, and the weight loss curve was recorded.

### 2.7. Effect of pH and Salt Ion Concentration on Solubility

A protein concentration of 10 mg/mL of SMP solution was prepared with ultrapure water as the solvent. The pH of the solution was adjusted to 3, 4, 5, 6, 7, 8, 9, 10, and 11 with 1 M NaOH and 1 M HCl, to form the pH group. A protein concentration of 10 mg/mL SMP solutions were prepared using NaCl solutions with concentrations of 1.0, 0.9, 0.8, 0.7, 0.6, 0.5, 0.4, 0.3, 0.2, and 0.1 mol/L as solvents, to form the salt ion concentration group. Each group was homogenized in a thermostatic mixer at 25 °C and 800 r/min for 15 min. After removal, the samples were centrifuged at 6000 rpm for 10 min, and the supernatant was retained. The concentration of the supernatant was determined using the Coomassie Brilliant Blue method. Solubility was calculated according to the following formula [30]:

(3) $$Solubility(\%)=\frac{C1}{C2}\times 100\%$$

Among these, *C*1 represents the protein concentration in the supernatant (mg/mL), and *C*2 denotes the initial protein concentration of the sample (mg/mL).

### 2.8. Effect of pH Value and Salt Ion Concentration on Foaming Performance

This assay was adapted from the method of Du et al. with minor modifications [28,31]. SMP solutions for the pH group and salt ion concentration group were prepared separately using the method described in Section 2.6. Using a high-speed disperser (T25 easy clean digital, IKA, Stuttgart, Baden-Württemberg, Germany), the protein solutions were homogenized sequentially at 10,000 r/min for 2 min. After homogenization, the samples were promptly transferred to 100 mL transparent glass graduated cylinders, and the volumes of the protein solutions before and after homogenization were recorded. The solutions were allowed to stand for 30 min, and their volumes were recorded again. Foaming capacity (FC) and foaming stability (FS) were calculated according to the following formula:

(4) $$FC\left(\%\right)=\frac{V_{1}-V_{0}}{V_{0}}\times 100\%$$

(5) $$FS\left(\%\right)=\frac{V_{2}-V_{0}}{V_{1}-V_{0}}\times 100\%$$

where *V*_0_ is the initial volume of the solution (mL), *V*_1_ is the volume of the solution after homogenization (mL), and *V*_2_ is the volume of the solution after standing for 30 min (mL).

### 2.9. Effect of pH Value and Salt Ion Concentration on Emulsification Properties

A protein concentration of 1 mg/mL of SMP solution was prepared using phosphate-buffered saline (PBS, 0.01 mol/L, pH 7.0) as the solvent. The pH of the solution was adjusted to 3, 4, 5, 6, 7, 8, 9, 10, and 11 with 1 M NaOH and 1 M HCl, respectively, to form the various pH-convention group. The preparation method for the salt-convention group was the same as described in Section 2.6. An amount of 5 mL of soybean oil was added to each solution, and the mixtures were homogenized for 1 min using a high-speed disperser at 12,000 rpm. From the same position at the bottom, 100 μL of the above solution was transferred into 5 mL of 0.1% (*w*/*v*) SDS solution. After 10 min, another 100 μL of the same solution was transferred into 5 mL of 0.1% (*w*/*v*) SDS solution using the same method. An aliquot of 200 μL of the mixed solution was taken, and the absorbance at 500 nm was measured using a microplate reader (SpectraMax^®^ iD5, Molecular Devices, Sunnyvale, CA, USA). The emulsifying activity index (EAI) and emulsification stability (ES) were calculated according to the following formula [32,33]:

(6) $$EAI(m^{2}/g)=\frac{2\times 2.303\times A_{0}\times DF}{C\times \Phi \times 10,000}\;$$

(7) $$ES\left(\%\right)=\frac{EAI(0\;min)}{EAI(10\;min)}\times 100\%$$

where *A*_0_ is the absorbance at 500 nm, *DF* is the dilution factor, *C* is the initial protein concentration (g/mL), and *Φ* is the volume fraction of oil in the emulsion.

### 2.10. Statistical Analysis

All the experimental data were processed using Origin 2021 (OriginLab Inc., Northampton, MA, USA). Statistical analysis was performed using SPSS Statistics 27 (IBM Inc., Armonk, NY, USA), and the one-way ANOVA was used to determine the significance of differences between means. The results are presented as the mean ± standard error (SE), with *n* = 3.

## 3. Results and Discussion

### 3.1. Isoelectric Point

**Figure 1:**
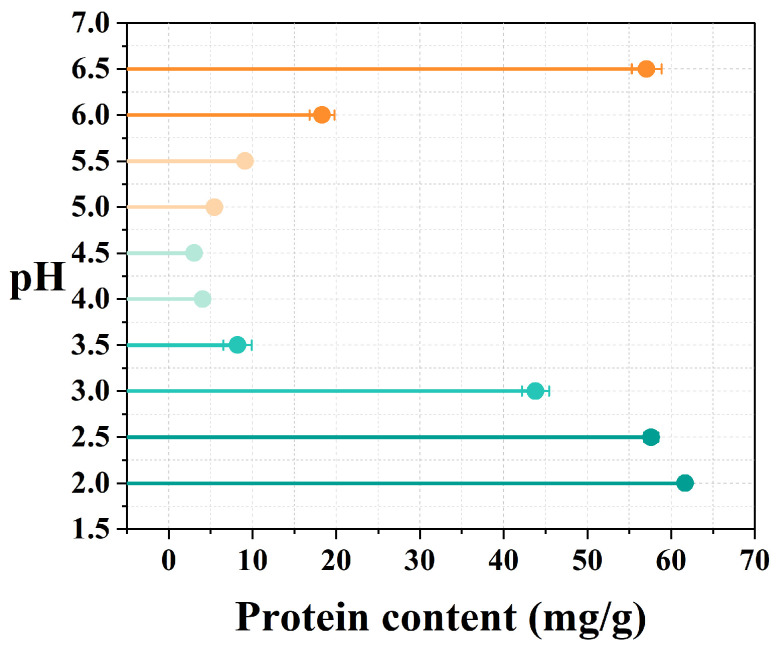
Measurement of protein isoelectric point of safflower seed meal.

As shown in Figure 1, the protein content of safflower seed meal decreased initially and then increased within a pH ranging from 2.0 to 6.5. Proteins are amphiprotic electrolytes. At their isoelectric point, proteins have an equal tendency to dissociate into positive and negative ions, which renders them neutral with a net charge of zero [34]. At this stage, the intermolecular forces experienced by protein molecules in solution are weakened due to the absence of mutual repulsion from identical charges, causing the particles to readily collide and aggregate, thereby resulting in precipitation. Therefore, at the isoelectric point, protein solubility is minimal and proteins are highly prone to forming precipitates [35]. At pH 4.5, the protein content was at its lowest, indicating minimal protein solubility in the safflower seed meal supernatant under these conditions. This corresponded to the isoelectric point of the protein. At pH values above 4.5 (the isoelectric point of the protein), the negative charges on the protein surfaces increased, leading to heightened electrostatic repulsion between them; this results in proteins being distributed more uniformly in water, thereby improving their solubility. Conversely, at pH values below 4.5, the positive surface charges of proteins increased due to the action of H^+^, which leads to enhanced solubility. As the pH approached the isoelectric point from either side, protein solubility decreased and the amount of protein in the supernatant gradually decreased. This experiment determined the isoelectric point of safflower seed meal protein (SMP) to be pH 4.5. Thereafter, this pH served as the acid precipitation point for the subsequent alkali-soluble, acid-precipitable SMP extraction method.

### 3.2. Protein Extraction Rate

Safflower seed contains 30–40% oil of which linoleic acid accounts for about 80%, and also contains a small amount of flavonoids and quinone chalcone safflower polyphenols. Linoleic acid is easily oxidized to produce peroxide, which covalently binds to proteins to cause denaturation [36]. Polyphenols will also covalently combine with protein amino groups and sulfhydryl groups to form insoluble substances, both of which will reduce the extraction rate of proteins [37]. Therefore, it is necessary to defat before extracting the protein of safflower seed meal. At the same time, the isoelectric point precipitation method is used to effectively inhibit the oxidation of linoleic acid, and quickly separate the protein from soluble components such as polysaccharides and free amino acids, so as to improve the purification efficiency. According to China’s GB 5009.5-2016 “National Food Safety Standard: Determination of Protein in Food” [38], the conversion factor for protein in safflower seeds is 5.30. Protein content was calculated by multiplying the nitrogen content by this factor, as shown in Table 1. The protein content in SMP was approximately 85%, while that in defatted safflower seed meal was about 54%. The results indicated that safflower seed meal contains substantial protein content after the hulls and skins have been removed, which was significantly higher than that of wheat, corn and other cereal proteins [39]. At the same time, safflower seed meal protein, as a typical agricultural by-product, relies on the characteristics of wide adaptability of safflower planting and a stable supply of raw materials, and the cost of raw materials is much lower than that of mainstream plant proteins such as soybeans and peas. It is a potential green and sustainable plant protein alternative source in the food industry. Making full use of these resources opens up new possibilities for the high-value use of safflower, an important cash crop. After the protein content of defatted safflower seed meal and SMP was determined, these values were entered into Formula (1), and the protein extraction rate of safflower seed meal was calculated to be 55%.

### 3.3. SDS-PAGE

**Figure 2:**
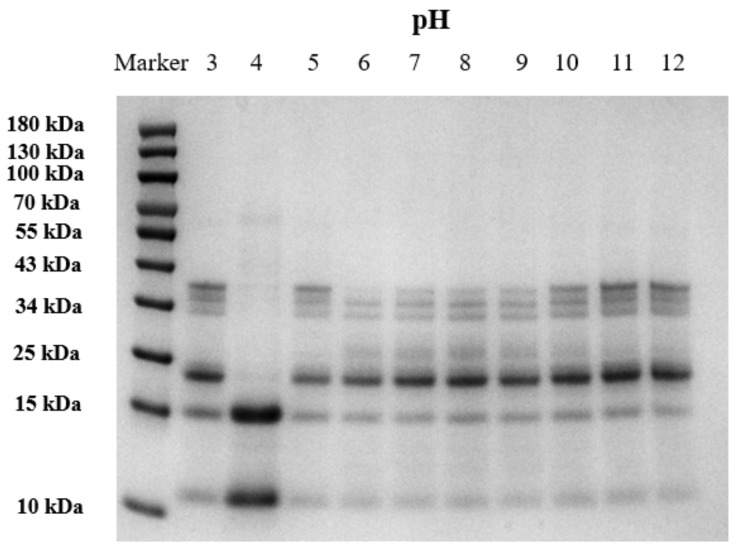
SDS-PAGE of safflower seed meal proteins after different pH interventions.

The results of SDS-PAGE revealed the molecular weights of the major components of the soluble SMP at different pH. Figure 2 shows the molecular weight distribution of SMP under different pH conditions. The molecular weight range of SMPs generally fell within 10–70 kilodaltons (kDa), with most proteins located between 10 and 43 kDa. Among these, peptides in the 20–29 kDa and 29–44 kDa ranges may be associated with the acidic and basic subunits of glycine [40,41]. Protein subunits of 15 kDa and below may be associated with albumin [42]. The distribution of SMP was essentially similar across other pH values, except at pH 4. At a pH of 4, the quantity of proteins with a molecular weight of around 10–15 kDa increased, while the quantity of proteins with other molecular weights decreased. This may occur because this pH approaches the isoelectric point of SMP, where changes in the hydrophilic–hydrophobic interactions between protein molecules promote aggregation and precipitation. The retained proteins are predominantly the water-soluble albumin, which leads to alterations in the soluble protein fraction.

### 3.4. Amino Acid Composition Analysis

Tryptophan contains an indole ring structure, which will be completely destroyed during the conventional acid hydrolysis pre-treatment process, so it cannot be detected by an automatic analyzer after standard acid hydrolysis. Seventeen amino acids except tryptophan were quantified. The amino acid composition and content of defatted safflower seed meal and SMP are shown in Table 2. Based on the known literature indicating that safflower seeds contain approximately 0.3% tryptophan, it is evident that safflower seed meal and safflower seed meal protein possess a complete profile of essential amino acids [43]. Except for threonine, the content of all other essential amino acids in both was higher than the Food and Agriculture Organization/World Health Organization (FAO/WHO) recommended levels for adults [44,45]. Moreover, both exhibited relatively high levels of glutamic acid (Glu) and arginine (Arg). Glu is a precursor for glutathione synthesis and can be used to synthesize proteins and other non-essential amino acids. It plays a crucial role in mediating excitatory synaptic transmission, maintaining intestinal functional integrity, and regulating energy homeostasis [46]; Arg is a functional amino acid that participates in the ornithine cycle within the human body, facilitating the conversion of ammonia into urea and thereby reducing blood ammonia levels [47]. The essential amino acids in SMP account for 31.79% of total amino acids, slightly higher than soy protein, brown rice protein, and pea protein, and significantly greater than oat protein, lupin protein, and wheat protein [48]. Although SMP has a relatively low tryptophan content, its lysine (Lys) content is higher than that of soy protein and pea protein [48]. Lys deficiency may lead to connective tissue disease, impaired fat function, acid metabolism, anemia and protein-energy malnutrition.

To evaluate the nutritional value of SMP and identify its limiting amino acids, an amino acid score (AAS) was calculated for its essential amino acids. The results are presented in Table 3. The AAS ratio is defined as the proportion of a specific essential amino acid in a protein compared to the corresponding amino acid content in the ideal model proposed by the WHO/FAO [49,50]. The closer the ratio is to 100, the higher the protein’s nutritional value. The amino acid with the lowest AAS value is known as the “first limiting amino acid” [51]. These limiting amino acids affect the utilization efficiency of other essential amino acids in the body and determine the quality of the protein to a certain extent. Based on the AAS values, all scores except for lysine (Lys) exceeded 60 points. The primary limiting amino acid in defatted safflower seed meal was Lys, followed by leucine (Leu). In SMP, Lys was the primary limiting amino acid, followed by isoleucine (Ile).

### 3.5. Thermogravimetric Analysis

**Figure 3:**
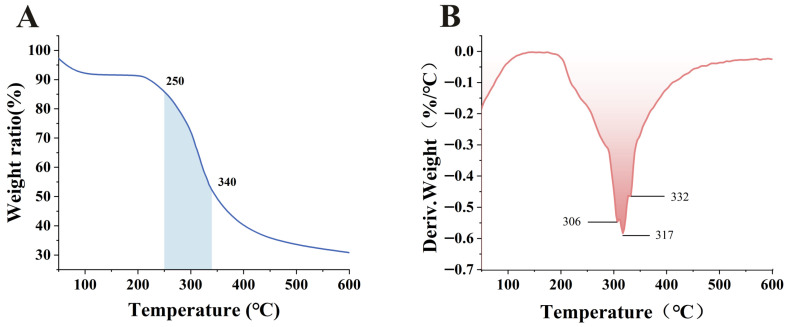
Thermogravimetric analysis curves (**A**) and corresponding derivative thermogravimetric analysis curves (**B**) for safflower seed meal proteins.

Thermal studies of proteins help determine their temperature-dependent behavior before significant thermal degradation occurs, which is particularly crucial for ensuring proteins retain their functionality during subsequent cooking, baking, or other heat treatments [52,53]. Figure 3A showed the results of the thermogravimetric analysis (TGA) performed to evaluate the thermal stability of SMP. The derivative thermogravimetric (DTG) curve is obtained by taking the first derivative of the TGA curve with respect to temperature, and it physically represents the relationship between weight loss rate and temperature [54]. This provides a more effective means of analyzing the thermal stability of the sample, as illustrated in Figure 3B. The SMP exhibited two stages of thermogravimetric loss. The first stage occurred between 50 and 100 °C and was primarily characterized by the evaporation of retained water and other volatile compounds within the SMP. The second stage occurred between 250 and 340 °C and was characterized by rapid degradation and significant mass loss during thermal decomposition. This is associated with the disruption of intramolecular and intermolecular interactions (such as hydrogen bonds, van der Waals forces and electrostatic interactions) at the protein’s melting point, leading to volatilization [55]. The peak loss temperatures in the second stage were 306, 317 and 332 °C. Of these, 317 °C was the maximum weight loss temperature for SMP. The thermal stability of SMP is comparable to that of most legume proteins, such as pea protein and lentil protein, making it suitable for food processing methods requiring high temperatures [56].

### 3.6. Solubility

**Figure 4:**
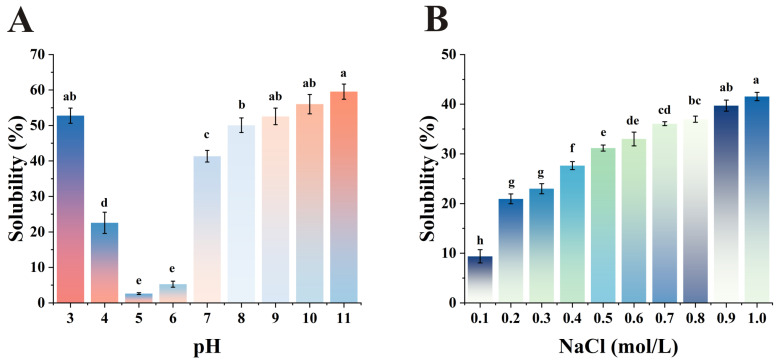
Effects of pH (**A**) and NaCl concentration (**B**) on safflower seed meal protein solubility. abcdefgh: Means labeled with the same letter are not significantly different (*p* < 0.05); *n* = 3.

The solubility of proteins is directly linked to many important functional properties and plays a crucial role in enhancing processing efficiency [57]. As shown in Figure 4A, the solubility of SMP at pH 3 to 11 could be divided into three distinct regions: acidic, near the isoelectric point and alkaline region. At a pH of 5, solubility was minimal at just 2.19%. This was because the pH was close to the isoelectric point of safflower seed meal protein (pH = 4.5), causing the protein particles to collide and aggregate readily, resulting in precipitation. Consequently, the soluble protein content of the solution decreased, resulting in reduced solubility. This result is similar to the report of mungbean, chickpea flours and peanut [24]. The maximum solubility values were observed at pH 3 (52.45%) and pH 11 (55.96%) at acidic and alkaline pH values, respectively. At high pH values, the conversion of carboxyl groups (-COOH) may have been promoted, thereby weakening the protein’s hydrogen bonds and van der Waals forces. This process facilitates structural unfolding and enhances hydration capacity [58,59]. The dissolution curve of SMP under different pH conditions exhibits a U-shaped pattern, similar to most plant proteins. Under neutral conditions, SMP’s solubility is lower than that of soybean but higher than chickpea flours, mung beans, and faba beans [60].

As shown in Figure 4B, low concentrations of neutral salts can increase the solubility of proteins in water [61]. As the NaCl concentration gradually increased from 0.1 mol/L to 1.0 mol/L, the solubility of SMP increased accordingly. At 1.0 mol/L, solubility reached a maximum of 41.56% within the study range. This may be due to sodium (Na^+^) and chloride (Cl^−^) ions binding to amino acid residues, which causes protein molecules to repel each other, thereby increasing solubility [62]. The solubility of SMP can be improved in subsequent food processing by increasing the salt ion concentration appropriately. Although increasing the salt ion concentration is less effective than adjusting the pH in enhancing its solubility, its solubility under these conditions remains higher than that of rice protein [63].

### 3.7. Foaming Capacity and Foaming Stability

**Figure 5:**
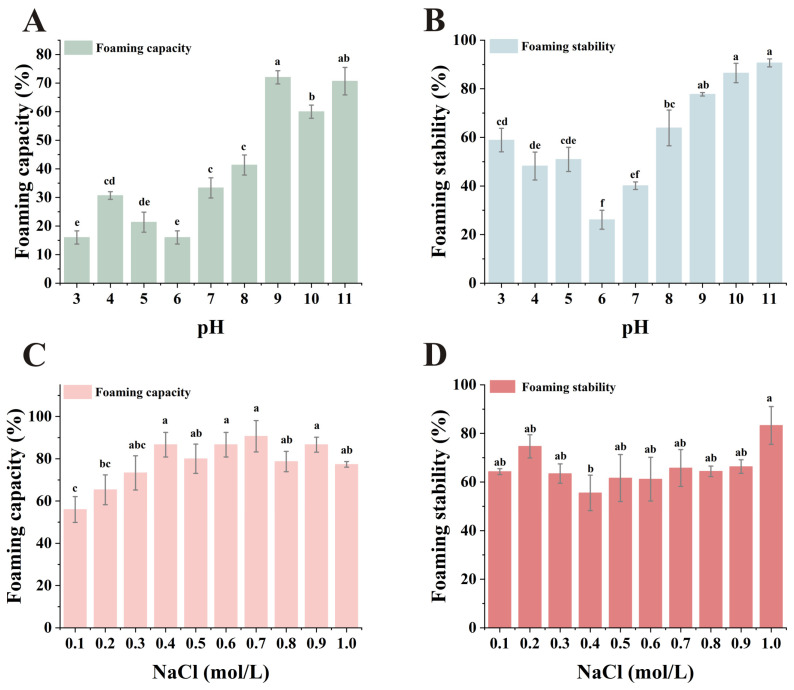
Effects of pH and NaCl concentration on foaming capacity (FC) and foaming stability (FS): (**A**) Effect of pH on FC; (**B**) Effect of pH on FS; (**C**) Effect of NaCl concentration on FC; (**D**) Effect of NaCl concentration on FS. abcdef: Means labeled with the same letter are not significantly different (*p* < 0.05); *n* = 3.

The foaming property of proteins refers to the strength with which a protein solution forms stable foam at the air–water interface. In the food industry, particularly in the processing of meat products, bread and pastries, the foaming property is an important characteristic and quality control indicator. As shown in Figure 5A,B, under acidic conditions near the isoelectric point of SMP, protein solubility decreased and electrostatic repulsion weakened, resulting in reduced foaming ability and foam stability. The foaming properties and foam stability of proteins increased under alkaline pH conditions. On one hand, high solubility may enhance the interaction between water and proteins, thereby promoting foam formation [64]. On the other hand, elevated pH increases the net charge carried by proteins, reducing hydrophobic interactions. This allows proteins to efficiently penetrate the water–air interface, envelop bubbles, and accelerate foam generation [65].

As shown in Figure 5C,D, foaming increased as the NaCl concentration rises from 0.1 mol/L to 0.4 mol/L. This can be attributed to the increased solubility of SMP at higher NaCl concentrations, since solubility is one of the key factors that influence foaming properties [66]. When the NaCl concentration exceeded 0.4 mol/L, the foaming properties did not show significant changes, probably because the added SMP had dissolved completely. At different NaCl concentrations, SMP’s foaming stability remained nearly consistent across all concentrations except at 1 mol/L, where it reached its peak.

SMP had lower FC under neutral conditions, but it was still higher than that of soybean and chickpea [60]. After adjusting the pH or regulating the concentration of salt ions, its FC was greatly improved, and it had good foaming performance as pea protein [60]. Although SMP has lower FS than pea protein, it can effectively stabilize the resulting foam [63]. Proteins exhibiting favorable foaming properties and foam stability can also serve as egg substitutes to enhance the softness and quality of baked goods.

### 3.8. Emulsifying Properties

**Figure 6:**
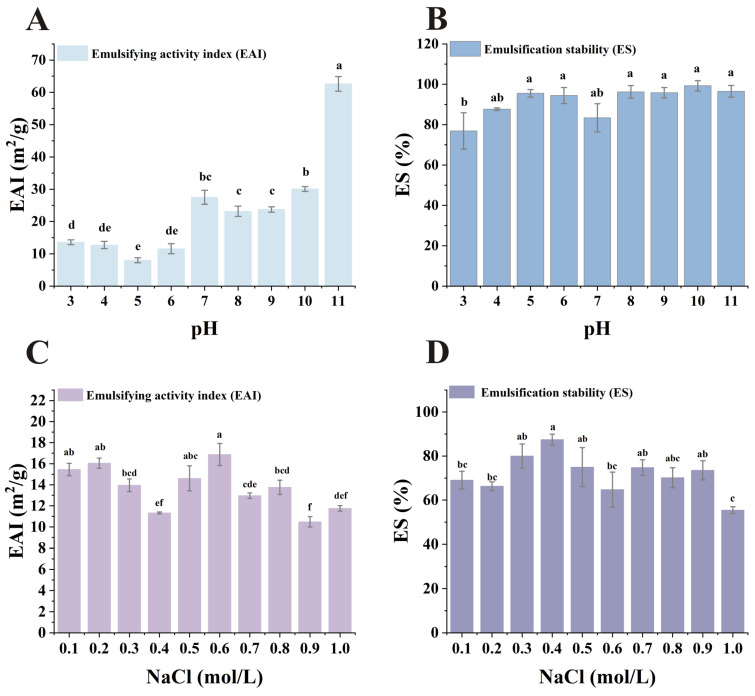
Effects of pH and NaCl concentration on emulsifying activity index (EAI) and emulsification stability (ES): (**A**) Effect of pH on EAI; (**B**) Effect of pH on ES; (**C**) Effect of NaCl concentration on EAI; (**D**) Effect of NaCl concentration on ES. abcdef: Means labeled with the same letter are not significantly different (*p* < 0.05); *n* = 3.

Emulsifying capacity reflects a sample’s ability to rapidly adsorb at the oil/water interface during emulsion formation by preventing flocculation and coalescence [65]. Emulsifying performance is quantified using the emulsifying activity index (EAI) and emulsification stability (ES) [67]. As shown in Figure 6A,B, the emulsifying activity index was low near the isoelectric point of SMP. Following treatment at different pH values, the emulsifying activity index increased to varying degrees, reaching a maximum at pH 11. This may be because, at the isoelectric point, the net charge of proteins can be minimized at that pH value. Higher surface net charges stabilize oil droplets and prevent emulsion droplet aggregation, aiding protein diffusion toward the oil–water interface and promoting more uniform dispersion, thereby exhibiting superior emulsifying properties [26,68]. At alkaline pH, the higher structure of the protein can be unfolded, the exposure of the hydrophobic amino acid side chain groups increases, and the surface hydrophobicity is enhanced, thereby increasing the emulsifying activity index of the protein [69]. Additionally, the pH dependence of emulsion volume can be explained by the fact that the properties of protein surfactants depend on the hydrophilic–lipophilic balance, which is influenced by pH [64,70]. The emulsifying stability of SMP remained relatively constant under different pH conditions. Near its isoelectric point, SMP’s EAI is comparable to most plant proteins; at pH conditions far from its isoelectric point, its EAI is higher than that of pea protein, chickpea protein, and rice dreg protein, but lower than that of peanut protein [24,32,71,72]. The emulsifying properties of SMP are superior to most plant proteins under alkaline conditions [60]. In summary, SMP exhibits excellent emulsifying performance.

NaCl can alter the surface charge of protein molecules, thereby weakening or enhancing the hydrophobic interactions of proteins, which in turn affects their emulsifying activity index and emulsion stability [33,73]. As shown in Figure 6C,D, emulsifying activity first decreased within the 0.1–0.4 mol/L range, then gradually increased within the 0.4–0.6 mol/L range, before decreasing again beyond 0.6 mol/L. SMP emulsion stability reached its peak at 0.4 mol/L. Below or above this concentration, the stability of the emulsion decreased to varying degrees. The effect of ionic strength on protein emulsifying activity is a complex phenomenon. Initially, increased ionic concentration may alter the hydrophilic/lipophilic balance by enhancing surface hydrophobicity; conversely, ions may also shield the net charge of proteins, thereby suppressing electrostatic repulsion and reducing emulsifying properties [74]. In the food industry, the optimal concentration of NaCl for SMP to exhibit its emulsifying activity is 0.5 or 0.6 mol/L, while the ideal concentration for achieving emulsifying stability is 0.4 mol/L. However, the method of enhancing SMP EAI and ES by adjusting salt ion concentration is less effective than adjusting pH.

### 3.9. Correlation Analysis

**Figure 7:**
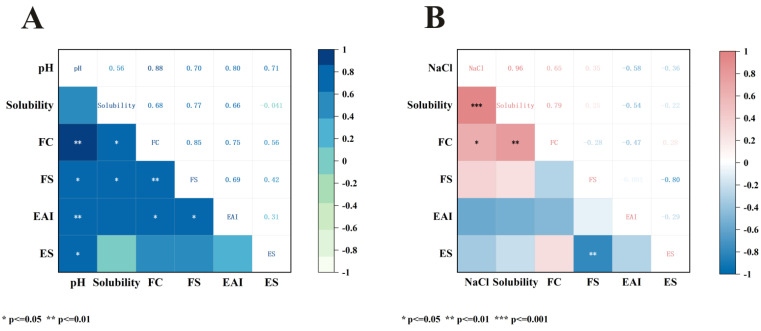
This heatmap shows the Pearson correlation coefficients between the functional properties of safflower seed meal protein and pH (**A**) and NaCl (**B**). EAI: emulsifying activity index; ESI: emulsifying stability index; FC: foam capacity; FS: foam stability.

Heatmaps visualize the correlation between different pH/NaCl concentrations and functionality. Correlation coefficients (r) close to 1 or −1 indicate strong positive or strong negative correlations, respectively. As shown in Figure 7, there was a positive correlation between pH and solubility (r = 0.56), FC (r = 0.88), FS (r = 0.70), EAI (r = 0.80) and ES (r = 0.71). The positive correlation between pH and solubility did not reach statistical significance, possibly due to the U-shaped relationship between the two variables. When the correlation coefficient was calculated from the isoelectric point, r = 0.91 (*p* ≤ 0.01). The above phenomena suggest that the pH level during subsequent processing is crucial for regulating the functional properties of SMP. NaCl exhibited positive correlations with solubility (r = 0.96), FC (r = 0.65), and FS (r = 0.35), and negative correlations with EAI (r = −0.58) and ES (−0.36). This may be because, at high ionic strengths, proteins tend to aggregate due to reduced net charge, causing the emulsion to become unstable through flocculation. Similar phenomena have been observed in other studies [62,75]. Notably, Figure 7A,B collectively demonstrate a significant positive correlation between solubility and foaming properties. These properties can be regulated by increasing solubility in subsequent protein processing.

## 4. Conclusions

This study involved an in-depth investigation of SMP, focusing on its physicochemical properties, such as its isoelectric point, amino acid composition and molecular weight distribution, as well as its processing characteristics, including solubility, emulsifying properties, foaming ability and foam stability. When proposing alternative plant-based protein sources for the food industry, it is essential to first consider the inherent protein content and extractability of raw materials. Simultaneously, the protein must have a balanced amino acid profile and favorable nutritional value. Furthermore, it should exhibit processability suitable for industrial production, such as solubility and foaming capacity. The findings of this study indicate that SMP not only features low raw material costs but also has an excellent protein content, desirable nutritional value, and multiple functional properties, making it a promising green and sustainable plant-based protein alternative for the food industry. With a protein content of around 54%, safflower seed meal is classified as a high-protein agricultural by-product. The SMP extracted from it has a protein content of around 86%, an extraction rate of 55% and an isoelectric point of pH 4.5. The molecular weight of SMP ranges from 10 to 70 kDa, with most of the proteins concentrated between 10 and 43 kDa. With a maximum weight loss temperature of 317 °C, SMP exhibited good thermal stability, making it suitable for subsequent food processing. The first limiting amino acid was lysine and the second was isoleucine. All seven of the other essential amino acids were detected in SMP, except for tryptophan, which was not tested. This indicates that SMP is a protein resource with potential nutritional value. Proteins that contain high levels of glutamic acid, such as wheat gluten and rice protein, tend to be poorly soluble in water. Research indicated that increasing the pH level and adding salt ions can improve the water solubility of SMP. Furthermore, SMP’s emulsifying properties, foaming ability and foam stability all showed a positive correlation with pH. Based on the determination results of various functional characteristics and SMP’s isoelectric point, the pH stability range for SMP is pH 7–11. Therefore, subsequent applications should prioritize this pH range to maximize its functional properties. When producing emulsification-related products such as chocolate, puff pastry and salad dressings, it is more effective to increase the pH value. SMP’s emulsifying properties can be utilized to emulsify and bind fats in meat products, thereby reducing fat and liquid losses in items such as sausages and meat patties. It can also be employed to replace egg yolks in salad dressings, producing dressings with physical characteristics similar to egg-based dressings. However, when processing foaming-related foods such as bread, pastries and ice cream, adding a salt ion concentration of 0.6–0.7 yields superior results. SMP’s excellent foaming properties make it suitable for use in baked goods, such as replacing egg whites or milk proteins in cakes and muffins. This shows that processing conditions are crucial in adapting protein properties for different uses. This study holds significant importance for the high-value utilization of agricultural by-products from cash crops and will provide valuable insights into the potential applications of SMP in the food industry.

## Figures and Tables

**Table 1:** Protein content of defatted safflower seed meal and safflower seed meal protein.

Sample	Group	Nitrogen Content (g/100 g)	Protein Content (g/100 g)	Average Protein Content (g/100 g)
Degreased safflower seed meal	1	10.263	54.394	54.380 ± 0.025
	2	10.267	54.415	
	3	10.251	54.330	
Safflower seed meal protein	1	16.150	85.595	85.652 ± 0.035
	2	16.159	85.643	
	3	16.173	85.717	

**Table 2:** Composition of amino acids and their contents.

Classification g·(100 g) − 1	Amino Acid Name	Degreased Safflower Seed Meal	Safflower Seed Meal Protein	FAO/WHO, 2007 [45] (Adult)
Essential amino acid	Lys	1.41 ± 0.15	2.39 ± 0.14	1.6
	Phe	3.29 ± 0.15	7.77 ± 0.17	1.9
	Met	0.94 ± 0.04	1.01 ± 0.04	1.7
	Thr	2.10 ± 0.13	1.51 ± 0.07	0.9
	Ile	2.14 ± 0.07	1.86 ± 0.10	1.3
	Leu	4.08 ± 0.09	3.80 ± 0.06	1.9
	Val	3.06 ± 0.03	3.67 ± 0.14	1.5
Semi-essential amino acids	His	2.14 ± 0.13	3.50 ± 0.07	1.6
	Arg	7.12 ± 0.06	8.55 ± 0.15	
Non-essential amino acid	Ser	2.20 ± 0.10	2.04 ± 0.05	
	Glu	15.5 ± 0.18	14.11 ± 0.35	
	Gly	3.15 ± 0.20	2.38 ± 0.16	
	Ala	3.63 ± 0.10	4.63 ± 0.14	
	Cys	0.64 ± 0.08	0.77 ± 0.06	
	Asp	6.22 ± 0.10	3.69 ± 0.18	
	Tyr	2.20 ± 0.08	4.21 ± 0.11	
	Pro	2.80 ± 0.09	3.33 ± 0.06	
	Total amino acids	62.62	69.24	

**Table 3:** Protein amino acid scoring scale.

Classification	Degreased Safflower Seed Meal	Safflower Seed Meal Protein	WHO/FAO Model g·(100 g) − 1
Thr	84.0	60.4	2.5
Lys	29.4	49.8	4.8
Ile	71.3	62.0	3.0
Val	76.5	91.8	4.0
Phe + Tyr	133.9	292.2	4.1
Leu	66.9	62.3	6.1
Met + Cys	68.9	77.4	2.3
His	133.8	218.8	1.6

## Data Availability

The original contributions presented in the study are included in the article; further inquiries can be directed to the corresponding authors.

## Abbreviations

The following abbreviations are used in this manuscript:

SMP	Safflower seed meal protein
AAS	Amino acid score
EAI	Emulsifying activity index
ES	Emulsification stability
FC	Foaming capacity
FS	Foaming Stability
TGA	Thermogravimetric analysis
DTG	Derivative thermogravimetric
